# Very High-Aspect-Ratio Polymeric Micropillars Made by Two-Photon Polymerization

**DOI:** 10.3390/mi14081602

**Published:** 2023-08-14

**Authors:** Keynaz Kamranikia, Sébastien Dominici, Marc Keller, Niklas Kube, Karine Mougin, Arnaud Spangenberg

**Affiliations:** 1 Institut de Science des Matériaux de Mulhouse (IS2M), CNRS-UMR 7361, Université de Haute-Alsace, 15 rue Jean Starcky, 68057 Mulhouse, France; keynaz.kamranikia@uha.fr (K.K.); sebastien.dominici@uha.fr (S.D.); marc.keller@uha.fr (M.K.); niklaskube@googlemail.com (N.K.); karine.mougin@uha.fr (K.M.); 2 Université de Strasbourg, 67000 Strasbourg, France

**Keywords:** high-aspect-ratio micropillar, two-photon polymerization, capillary force

## Abstract

Polymeric micropillars with a high-aspect-ratio (HAR) are of interest for a wide range of applications, including drug delivery and the micro-electro-mechanical field. While molding is the most common method for fabricating HAR microstructures, it is affected by challenges related to demolding the final structure. In this study, we present very HAR micropillars using two-photon polymerization (TPP), an established technique for creating complex 3D microstructures. Polymeric micropillars with HARs fabricated by TPP often shrink and collapse during the development process. This is due to the lack of mechanical stability of micropillars against capillary forces primarily acting during the fabrication process when the solvent evaporates. Here, we report different parameters that have been optimized to overcome the capillary force. These include surface modification of the substrate, fabrication parameters such as laser power, exposure time, the pitch distance between the pillars, and the length of the pillars. On account of adopting these techniques, we were able to fabricate micropillars with a very HAR up to 80.

## 1. Introduction

High-aspect-ratio (HAR), the ratio of height to lateral feature size, polymeric microstructures with defined size and shape, such as micropillars, are of interest for a variety of applications, including drug delivery [1,2,3], dry adhesives that simulate the fibrillar structure of gecko feet [4], surface wetting properties [5,6], and micro-electromechanical systems (MEMS) [7,8,9]. Because of their large surface area and well-shaped periodicity, micro/nanopillars have been used for a wide range of research studies in designing and exploring liquid-repellent surfaces [10], manipulating cells by controlling topographical environments [11], and their tunable optical properties [12]. There are various methods for fabricating HAR micropillars, such as etching, electrospinning, electrospraying, self-assembly, and templates. While comparative analysis of those approaches is out of the scope of the current research article, one can find a recent and relevant review on this aspect [13]. Among the different classes of materials used for HAR fabrication, microstructures made of polymers are favored for numerous reasons, including transparency, compliance, biocompatibility, and cost-effectiveness [14,15].

The most common fabrication method for HAR micropillars is molding [16,17,18]. For this method, multiple replicas are produced from master structures by double-molding polymer replica molds. The polymer materials include polydimethylsiloxane (PDMS) [19,20,21], photocurable polymers [22], and thermoplastics [23]. This technique improves fabrication throughput and changes the structure by deforming the elastic polymer replica mold [24]. Polymeric micro-nano structures with an aspect ratio up to 15 have been fabricated by molding with PDMS [25,26]. PDMS is the most common mold and the advantages are its biocompatibility, mechanical properties, and transparency; it is gas-permeable water-impermeable, relatively inexpensive, and can be prototyped quickly with high precision using simple procedures [27]. However, it remains a challenge to fabricate HAR micropillars with molding due to the multiple steps process and the need for bonding the microstructure after removing it from a mold. Furthermore, microstructures with special materials, multi-materials, or surface properties cannot be manufactured without post-print treatment [28].

The other common methods of HAR micropillar fabrication are X-ray lithography [29] and UV lithography [30] in which the HARs of up to 10 and 20 have been fabricated, respectively. These methods, however, are not cost-effective and have a low throughput [31].

Recent studies have investigated the use of the photoresist SU-8 for fabricating HAR microstructures since it exhibits stable mechanical, thermal, and chemical characteristics [32,33]. HARs up to 40 and 100 were obtained by UV processing of ultra-thick photoresist films [34,35] and drawing lithography [36], respectively. Additionally, HARs up to 50 [37] and 10 [38] were previously obtained with SU-8 using the TPP technique.

In recent decades, much attention has been paid to using direct laser 3D printing HAR microstructures such as TPP, which is the most flexible method for the fabrication of micropillars [3,12]. TPP is a well-established method for creating complex 3D micro-objects with stimuli-responsive and reconfigurable surface properties [39,40]. In addition, unlike other micropillar microfabrication methods, rapid prototyping technologies such as TPP do not require any clean room environment and complex geometry components can be produced faster while requiring less technical expertise [41]. In TPP, a femtosecond or picosecond pulsed laser is used. By applying a focused laser beam to photosensitive materials, two-photon absorption (TPA) triggers polymerization. This provides a nonlinear energy distribution that is centered at the laser’s focal point. In the TPP process, the photoinitiator molecules in photoresist begin polymerization when they absorb this energy at regions known as polymerization voxels, where the absorption energy exceeds a particular threshold of the resin, resulting in a polymerized 3D micro/nanostructure [42].

Although the TPP can generate micropillars, it faces a number of challenges. One of these challenges is capillary force, which can result in significant defects, such as deformation or collapse [43]. Capillary forces are typically generated due to the evaporation during the final step of the microstructure development process by a solvent [44,45,46,47]. It is possible to reduce capillary forces by altering the substrate by surface modification or using different drying methods [48]. Liquids with low surface tension, such as isopropyl alcohol (IPA), can reduce capillary forces, but this approach shows limited improvement for nanostructures with moderate aspect ratios [49,50]. Further increasing the aspect ratio can cause the same deflection, and IPA cannot prevent HAR nanostructure clustering. An effective method for drying is supercritical drying, which involves increasing the temperature and pressure of the environment beyond the critical point and causing the liquid to reach the supercritical phase [51]. Pattern collapse can be suppressed most effectively by reducing surface tension. Using a supercritical carbon dioxide (CO_2_) dryer for a final development process, which has no surface tension, can allow the formation of fine micropillar patterns. As a result, nanostructures do not experience capillary forces [48,49,52].

In this study, we demonstrate very HAR micropillars up to 80 by TPP with a height of ~53 µm and a distance pitch of 5 µm. Different parameters such as pitch distance and printing settings were optimized. In order to prevent capillary forces from creating clusters and causing pillars to fall and collapse, surface modification of the glass substrate is performed. Furthermore, we attempt to use supercritical CO_2_ drying to avoid the collapsed micropillars.

## 2. Materials and Methods

### 2.1. Materials

All of the chemicals were used as received and were not purified any further. Pentaerythritol triacrylate (PETA), ethanol ≥ 99.8%, and 4,4′-bis(diethylamino) benzophenone (DEABP) were obtained from Sigma Aldrich (St. Louis, MO, USA).

### 2.2. Preparation of Photoresist

An amount of 3 wt% of DEABP was added to 97 wt% of PETA at room temperature and these were mixed by using a magnetic stirrer for 10 min.

### 2.3. Two-Photon Polymerization 3D Printer

**Figure 1:**
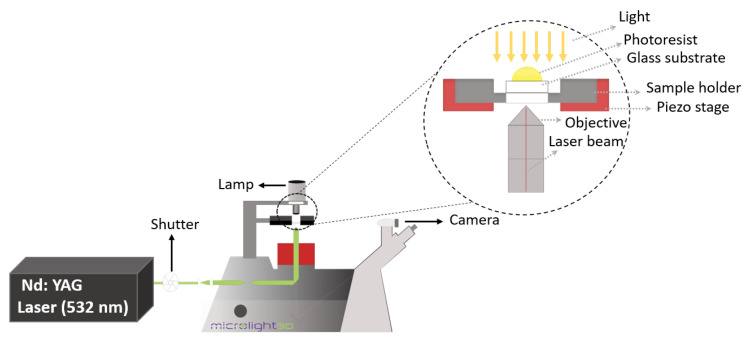
Scheme of the two-photon polymerization system. A sub-nanosecond pulsed Nd:YAG laser emits the 532 nm laser beam, which is directed through the shutter and the optical pathway to the objective that focuses the beam inside the photoresist. The lamp is placed on top of the sample and illuminates it from above. In order to take images or movies, a camera is part of the equipment. The close-up schematic shows a glass slide with a photoresist drop on it, which is attached to the sample holder. The laser beam hits the photoresist at the glass bottom.

A MicroFAB-3D printer (Microlight3D, La Tronche, France) was used for printing micropillars. The 532 nm laser beam, generated by a sub-nanosecond pulsed Nd:YAG laser, passes the shutter and the optical pathway to the objective which focuses the beam inside the photoresist. A lamp illuminates the sample from the top. A glass coverslip, on it a small drop of photoresist, is attached to the sample holder which is laying on the piezo stage. The laser beam hits the photoresist from the bottom of the glass. To protect the eyes of the operator, a filter is put on top of the sample stage (see Figure 1).

In order to measure the laser power, a Thorlabs PM100D device was used after setting the laser gain in the software Lithos, version 2.5.2.

### 2.4. Surface Modification on Glass Substrate

**Figure 2:**
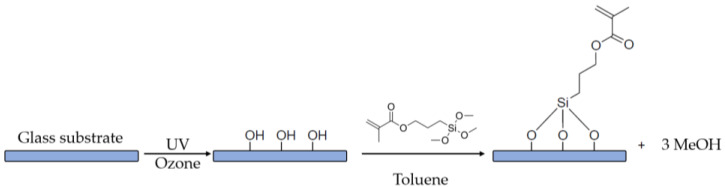
Reaction scheme of glass functionalization with 3-(trimethylsilyl)propyl methacrylate after UV/ozone treatment.

For the surface modification of the glass substrate, 22 mm × 22 mm glass slides were cleaned for 20 min in a BioForce Nanosciences UV/OZONE ProCleaner Plus. Afterwards the slides were immersed for 24 h in a functionalization solution consisting of 60 mL toluene and 1 mL of 3-(Trimethoxysilyl)-propyl methacrylate. The glass slides were taken out of the solution and washed with toluene and ethanol in order to remove any residues (see Figure 2).

### 2.5. Fabrication of Microstructures

**Figure 3:**
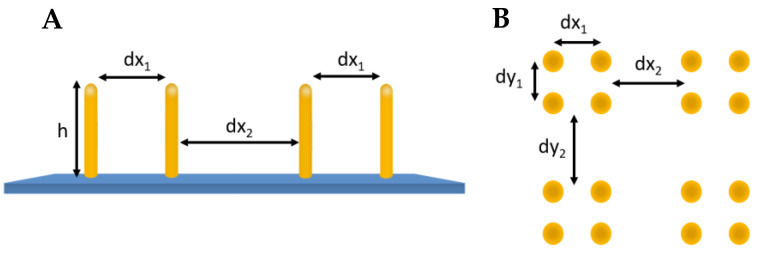
Arrangement of pillars in (**A**) side view and (**B**) top view.

The 3D model file of the pillars was generated as a .txt file using a Python 3.11.4 program. Parameters that could be adjusted include the distances between the pillars in the x-direction dx_1_ and dx_2_ and between the pillars in the y-direction dy_1_ and dy_2_. Furthermore, the number of pillars in x-direction N_-obj-x_ and in y-direction N_-obj-y_ and the height *h* of the pillars could be set as it is shown in Figure 3.

The Simpoly 4.4.5 application was used to slice the generated .txt file. It allows exporting a .tsk file that contains the printing pathways. Parameters for printing a structure by Simpoly 4.4.5 application are the printing quality, numerical aperture, type of objective (immersion or air), refractive index of the uncured resin, laser gain management, and path printing order (start from top or start from bottom). For all structures, the printing quality was set to ’intermediate’, which equals a voxel overlap ratio of 70%, and the numerical aperture was set to 0.65 Korr objective with Zeiss Plan Apochromat with 40× magnification.

In order to print the pillars, a droplet of photoresist was added to the functionalized glass substrate and attached to the sample stage which was laying on the piezo stage. The laser beam hits the photoresist from the bottom of the glass. All pillars were arranged in 12 × 12 arrays with pitch distances ranging from 1.5 µm to 5 µm. Exposure parameters were 2.70 mW and 10 ms for laser power and printing speed, respectively. In the development process, the structures were carefully rinsed using ethanol and subsequently dried using the supercritical CO_2_ dryer (Autosamdri 815, Series A automatic critical point dryer, tousimis, Rockville, MD, USA). Since supercritical CO_2_ has a low surface tension and high diffusivity, it has been used to prevent pattern collapse caused by capillary forces during drying. After rinsing with ethanol, the wet sample was transferred into the supercritical dryer chamber and filled with 15 mL of pure ethanol to ensure complete sample coverage. The liquid CO_2_ tank was opened and the purging time was set to 15 min. After several purge–flash cycles to completely remove the ethanol, the chamber was heated above the critical point of CO_2_ (31 °C, 1200 psi) and maintained for 10 min before slowly venting to the atmosphere.

### 2.6. Scanning Electron Microscopy

Scanning electron microscopy (SEM) of the generated TPP structures were examined from the top view and at tilt angles of 45° in the high-vacuum mode using a FEI, Quanta 400 SEM (FEI Company, Dawson Creek Drive, Hillsboro, OR, USA). The spot size and the accelerating voltage were set to 3.5 nm and 20 kV, respectively. Before taking SEM images, the samples were gold-coated using a Cressington 108 auto sputter coater (Microtonano, Haarlem, The Netherlands). Samples were fixed with clamps on a metallic sample holder. The aspect ratio of TPP micropillar structures was evaluated by measuring the pillar heights, pillar diameters, and the diameter at the pillar–substrate interface.

## 3. Results and Discussion

**Figure 4:**
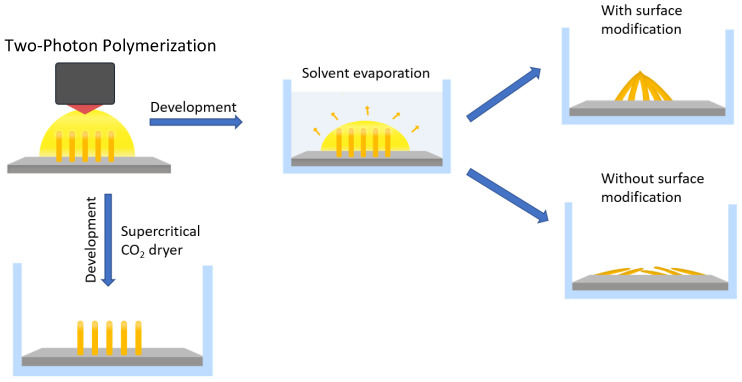
Schematic illustration of different development approaches. Upon two-photon polymerization via 532 nm laser beam exposure, the structures are fabricated. The first approach after printing the structure includes rinsing with the solvent and letting the sample dry in the air. By using this approach, a surface-modified glass substrate leads to the creation of a cluster and without it, the pillars fall down or collapse onto the glass surface. A second approach involves drying the printed structure using a supercritical CO_2_ dryer that prevents the pillars from collapsing or falling down.

The main idea of this study is to stabilize structural features before capillary forces are acting, as described in Section 2.5 and Figure 4.

### 3.1. Surface Modification of the Glass Substrate

The strategy to fabricate the HAR micropillar structure is schematically presented in Figure 4. Firstly, by focusing a 532 nm laser beam into a photoresist consisting of 97 wt% PETA and 3 wt% DEABP, polymer pillars were fabricated on a glass slide by a typical 3D laser printing process.

**Figure 5:**
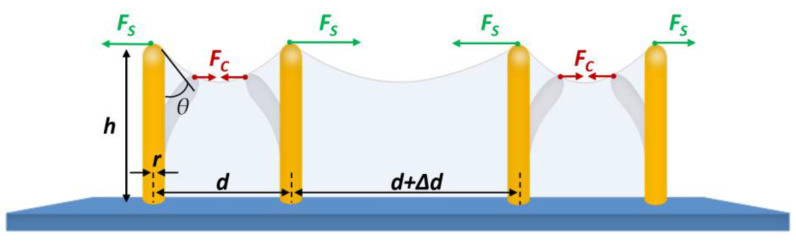
Schematic of the pillars’ arrangement with the acting forces, such as capillary force *F*_C_ and resistance force *F*_S_, during the sample development by using a solvent.

Following the laser printing, the sample was rinsed with ethanol during the development process to remove the unpolymerized photoresist. However, the adhesion between the glass substrate and the printed structure was insufficient after the development process, resulting in the printed structure falling and creating a cluster as shown in Figure 5. To prevent this, the glass coverslip was treated with silane functionalization. As shown in Figure 1, the hydroxide functional groups created on the surface of the glass substrate following UV/OZONE ProCleaner Plus treatment and Si–O–Si (siloxane) linkages occurred by immersing the glass into the 60 mL toluene and 1 mL of 3-(Trimethoxysilyl)-propyl methacrylate solution. This results in methacrylate groups being covalently bound on the glass surface [53].

**Figure 6:**
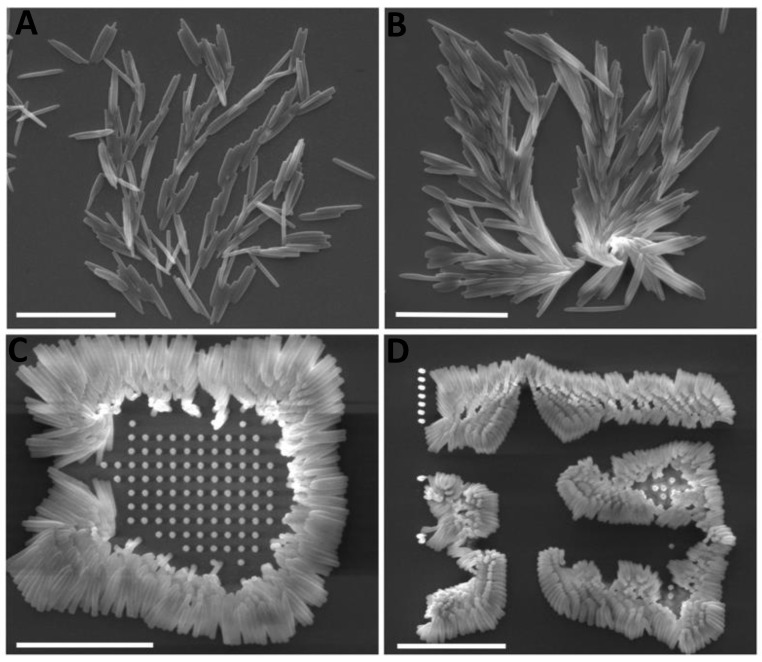
SEM images from the top view of the micropillars. Samples were fabricated by TPP by using an unfunctionalized glass substrate. All pillars were 3D printed on one unfunctionalized glass substrate with a laser power of 2.70 mW and with a height of 5 µm. Micropillars started to fall or create a cluster by decreasing the pitch distance from (**A**) 4 µm, (**B**) 3 µm, (**C**) 2 µm, and (**D**) 1.5 µm. All scale bars: 20 µm.

On the other hand, if the printed structures were dried under normal conditions after developing them with ethanol, the polymer pillars would be attracted towards each other by capillary-force self-assembly and create a cluster. With increasing pillar height, individual pillars bend and come into contact at the top. This new connection results from the evaporation-induced capillary force present in the subsequent development process that washes off the polymer that was not exposed to the laser. Upon evaporation of the solvent to the levels of the freestanding tips, a capillary force [54] *F*_C_ is the result between the neighboring pillars:

*F*_C_~*γ r*^2^ cos^2^*θ*/*d* (1)

which is proportional to the interfacial tension *γ* of the solvent, the square of the radius *r* of the pillars, and the squared cosine of the contact angle *θ* and inversely proportional to the pitch distance *d* between adjacent pillars, as shown in Figure 6.

Resistance to the capillary force is the restoring force [55]:

*F*_S_~*E r*^4^ *d*/*h*^3^ (2)

where *E* is Young’s modulus and *h* is the pillar height. There is a critical distance *d_c_* below which neighboring pillars can be assembled or otherwise remain upright when the capillary force and the restoring force are balanced. When *d* is lower than *d_c_*, neighboring pillars can remain in contact by capillary forces *F*_C_ during solvent evaporation or by short-range van der Waals forces *F*_V_ in the air for a given pillar height [54].

In addition, when the height is too high, the capillary force is so considerable that the pillars collapse into unordered structures and create clusters as it is shown in Figure 6. Conversely, as long as the height of the pillars is under a certain value, the capillary force will not overcome the standing force; therefore, the pillars will not collapse.

The distance pitch of micropillars influences the role of the capillary force after the structure is developed with the solvent. As the distance pitch between two pillars decreases, the chance of collapse increases. Figure 6 shows micropillars with different distance pitches printed on a normal glass substrate from 4 µm to 1.5 µm. The non-functionalized glass substrate resulted in micropillars falling and collapsing from the structures with higher and smaller pitch, respectively. Due to the stronger capillary force on the edges of the printed structure, pillars at the edges are more likely to collapse.

**Figure 7:**
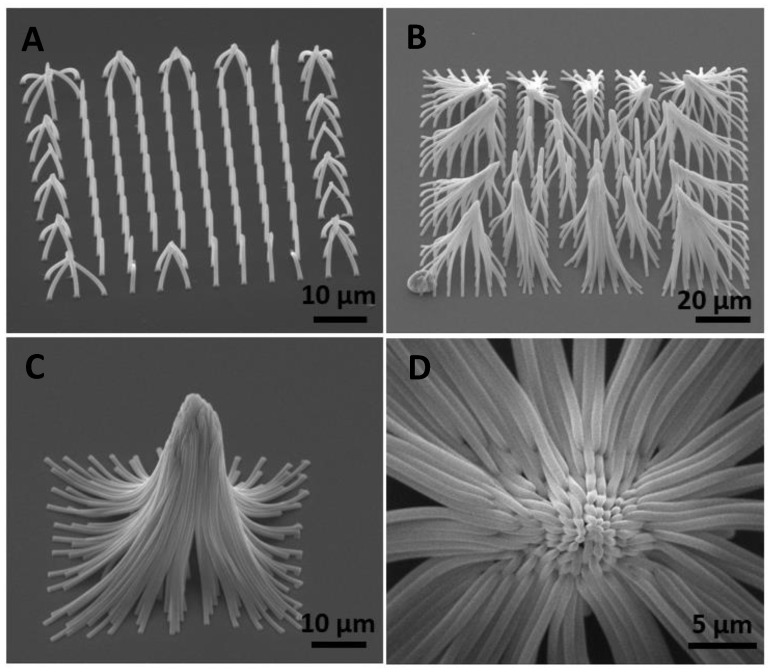
SEM images of micropillars fabricated by TPP on a glass coverslip with silane functionalization. Side view images of micropillars with the capillary-force self-assembly with the height of (**A**) 13 µm, (**B**) 37 µm, and (**C**) 55 µm. (**D**) Top view image of micropillars with a height of 55 µm. All micropillars were fabricated with the distance pitch of 5 µm. The sample stage is tilted by 45° for images (**A**–**C**) and −0.1° for image (**D**).

The micropillars shown in Figure 7 were fabricated by applying silane functionalization to a glass substrate. Once the structures have been rinsed with ethanol, pillars with aspect ratios greater than 10 collapsed due to the capillary force.

### 3.2. Very HAR Micropillars with Supercritical CO_2_ Dryer

Polymeric micropillars with 97 wt% PETA and 3 wt% DEABP were fabricated using a two-photon microfabrication system. The resultant microstructure was carefully rinsed with ethanol to remove the unsolidified photopolymer. The sample was immediately put into the chamber of the supercritical apparatus (Autosamdri 815, Series A automatic critical point dryer, tousimis, Rockville, MD, USA) which was immersed in 15 mL of pure ethanol. In this way, the surface was kept wet and the solvent was prevented from evaporating. The liquid CO_2_ tank was opened and the purging time was set to 15 min. After filling the chamber with supercritical CO_2_ and several purge–flash cycles to completely remove ethanol, the chamber was heated above the critical point of CO_2_ with a temperature of 31 °C and pressure of 1200 psi and maintained for 10 min before slowly venting to the atmosphere. The absence of surface tension during the supercritical CO_2_ drying allowed for recording of the fine micropillar patterns. All micropillars were arranged in 12 × 12 arrays with pitch distances of 5 µm. The laser power was set to 2.76 mW and the exposure time was set to 10 ms in Z direction. The dimensions of the micropillars were determined using the SEM images. The pillar heights and their diameters are listed in Table 1.

**Figure 8:**
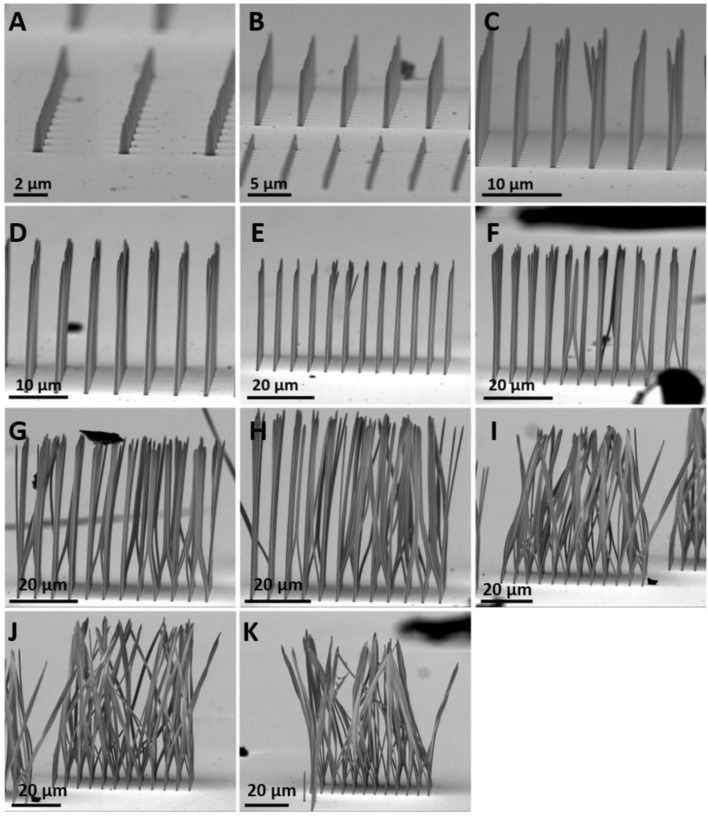
SEM images of micropillars with aspect ratio of (**A**) 3.8, (**B**) 10.23, (**C**) 22.33, (**D**) 33, (**E**) 44.27, (**F**) 57.71, (**G**) 62.67, (**H**) 80.29, (**I**) 83.7, (**J**) 90.09 and (**K**) 101, respectively. All pillars were arranged in 12 × 12 arrays with a pitch distance of 5 µm. The sample stage is tilted by 90°.

Figure 8 shows SEM images with an approximate tilting of 90° of micropillars with different aspect ratios. The aspect ratio of the micropillar was measured by dividing the height of the pillar by its diameter h/2r (see Figure 5). Due to the instability of pillars over 30 µm in height and less than 1 µm in diameter, micropillars with aspect ratios up to 40 did not remain completely upright. The very HAR of 80 that is shown in Figure 8H was obtained in this study. To the best of our knowledge, this is the highest aspect ratio of polymeric micropillars obtained to date using the TPP technique. Micropillars with a height greater than 50 µm can be observed in Figure 8H–K. The SEM images show that most of the micropillars are bent after development by the supercritical CO_2_ dryer. These micropillars have HARs of 80, 83, 90, and 101, respectively. In the supercritical CO_2_ dryer, due to the absence of surface tension, *γ* = 0 and thus resulted in *F*_C_ = 0 (see Equation (1)). This result indicates that the bending of pillars that are shown in Figure 8F–K are not provoked by the capillary force. It is believed that the bending is due to the turbulences induced by the introduction of liquid CO_2_ into the chamber to replace ethanol. These turbulences can cause pillars with a high height (over 40 µm) to bend. Due to the non-stand-up micropillars, the average height of all structures was not measured; the approximate numbers of the diameters and heights are listed in Table 1. Additionally, the three arrays of pillars in Figure 8A–E are properly defined and no collapsing can be observed between them.

Compared to the conventional HAR fabrication techniques, TPP solves limitations associated with other methods, such as defects in appearance, long solvent evaporation times, and multi-step processes [56]. Moreover, TPP is now seen as a scalable micro-additive manufacturing process which is compatible with many classes of materials. Therefore, one can envision TPP as a relevant alternative to produce very HAR micropillars [57]. Indeed, many applications are possible with HAR micropillars, such as smart display and sensing, self-powered systems and soft robotics, droplet transport, and surface reversible switchable wettability [13]. However, the supercritical CO_2_ dryer seems not to be an appropriate method for high-throughput fabrication processes as required by the semiconductor industry.

## 4. Conclusions

In this study, we have demonstrated micropillars with a very HAR of up to 80 that were fabricated using TPP. The key was using surface modified glass substrates for the fabrication, as well as supercritical CO_2_ drying. The use of supercritical drying allowed HAR micropillars to be fabricated. It was shown that by adjusting a pitch distance of 5 µm between the micropillars and using 2.76 mW laser power, the free-standing micropillars with a maximum 52 µm height was achievable. It is believed that printing HAR micropillars by applying the TPP technique will find numerous applications in droplet transport, smart display and sensing, surface modification, and the development of new MEMS in future.

## Figures and Tables

**Table 1:** The aspect ratio of micropillars.

Sample	L_corr_/µm	Ø/µm	Aspect Ratio
A	1.90 ± 0.07	0.50 ± 0.01	3.80
B	6.75 ± 0.28	0.66 ± 0.05	10.23
C	14.29 ± 0.20	0.64 ± 0.03	22.33
D	21.75 ± 0.13	0.66 ± 0.03	33.00
E	29.22 ± 0.16	0.66 ± 0.04	44.27
F	37.51 ± 0.24	0.65 ± 0.05	57.71
G	45.12 ± 0.11	0.72 ± 0.01	62.67
H	52.99 ± 0.14	0.66 ± 0.04	80.29
I	59.04 ^1^	0.71 ^1^	83.70
J	57.24 ^1^	0.74 ^1^	90.09
K	73.64 ^1^	0.73 ^1^	101

^1^ Due to the non-straight pillars, the average height could not be obtained.

## Data Availability

Not applicable.

## References

[1] McAllister D.V., Wang P.M., Davis S.P., Park J.-H., Canatella P.J., Allen M.G., Prausnitz M.R. (2003). Microfabricated needles for transdermal delivery of macromolecules and nanoparticles: Fabrication methods and transport studies. Proc. Natl. Acad. Sci. USA.

[2] Park J.-H., Allen M.G., Prausnitz M.R. (2005). Biodegradable polymer microneedles: Fabrication, mechanics and transdermal drug delivery. J. Control. Release.

[3] Rad Z.F., Prewett P.D., Davies G.J. (2021). High-resolution two-photon polymerization: The most versatile technique for the fabrication of microneedle arrays. Microsyst. Nanoeng..

[4] Geim A.K., Dubonos S.V., Grigorieva I.V., Novoselov K.S., Zhukov A.A., Shapoval S.Y. (2003). Microfabricated adhesive mimicking gecko foot-hair. Nat. Mater..

[5] Suh K., Jon S. (2005). Control over Wettability of Polyethylene Glycol Surfaces Using Capillary Lithography. Langmuir.

[6] Jeong H.E., Lee S.H., Kim J.K., Suh K.Y. (2006). Nanoengineered multiscale hierarchical structures with tailored wetting properties. Langmuir.

[7] Madou M.J. (2002). Fundamentals of Microfabrication: The Science of Miniaturization.

[8] Kim K., Park S., Lee J.-B., Manohara H., Desta Y., Murphy M., Ahn C.H. (2002). Rapid replication of polymeric and metallic high aspect ratio microstructures using PDMS and LIGA technology. Microsyst. Technol..

[9] Cadarso V.J., Pfeiffer K., Ostrzinski U., Bureau J.B., Racine G.A., Voigt A., Gruetzner G., Brugger J. (2011). Direct writing laser of high aspect ratio epoxy microstructures. J. Micromech. Microeng..

[10] Wong T.-S., Kang S.H., Tang S.K.Y., Smythe E.J., Hatton B.D., Grinthal A., Aizenberg J. (2011). Bioinspired self-repairing slippery surfaces with pressure-stable omniphobicity. Nature.

[11] Buch-Månson N., Spangenberg A., Gomez L.P.C., Malval J.-P., Soppera O., Martinez K.L. (2017). Rapid prototyping of polymeric nanopillars by 3D direct laser writing for controlling cell behavior. Sci. Rep..

[12] Purtov J., Rogin P., Verch A., Johansen V.E., Hensel R. (2019). Nanopillar diffraction gratings by two-photon lithography. Nanomaterials.

[13] Li C., Yang J., He W., Xiong M., Niu X., Li X., Yu D.-G. (2023). A review on fabrication and application of tunable hybrid micro–nano array surfaces. Adv. Mater. Interfaces.

[14] Colombo P., Mera G., Riedel R., Sorarù G.D. (2013). Polymer-derived ceramics: 40 years of research and innovation in advanced ceramics. Ceramics Science and Technology.

[15] Manz A., Becker H. (1998). Microsystem Technology in Chemistry and Life Science.

[16] Higgins S.G., Becce M., Belessiotis-Richards A., Seong H., Sero J.E., Stevens M.M. (2020). High-aspect-ratio nanostructured surfaces as biological metamaterials. Adv. Mater..

[17] Chen C.-M., Yang S. (2014). Directed water shedding on high-aspect-ratio shape memory polymer micropillar arrays. Adv. Mater..

[18] Chen C.-M., Chiang C.-L., Lai C.-L., Xie T., Yang S. (2013). Buckling-based strong dry adhesives via interlocking. Adv. Funct. Mater..

[19] Shao G., Wu J., Cai Z., Wang W. (2012). Fabrication of elastomeric high-aspect-ratio microstructures using polydimethylsiloxane (PDMS) double casting technique. Sens. Actuators A Phys..

[20] Block I.D., Chan L.L., Cunningham B.T. (2007). Large-area submicron replica molding of porous low-k dielectric films and application to photonic crystal biosensor fabrication. Microelectron. Eng..

[21] Shinohara H., Goto H., Kasahara T., Mizuno J. (2013). Fabrication of a polymer high-aspect-ratio pillar array using UV imprinting. Micromachines.

[22] Shinohara H., Tashiro T., Ookawa T., Nishihara H. (2012). High-throughput UV nanoimprint process using flexible resin mold for high-brightness light-emitting diodes. IEEJ Trans. Sens. Micromachines.

[23] Shibazaki T., Shinohara H., Hirasawa T., Sakai N., Taniguchi J., Mizuno J., Shoji S. (2010). Anti-sticking curing of fluorinated polymers for improvement of mold releasability. J. Photopolym. Sci. Technol..

[24] Copic D., Park S.J., Tawfick S., Volder M.F.L.D., Hart A.J. (2011). Fabrication of high-aspect-ratio polymer microstructures and hierarchical textures using carbon nanotube composite master molds. Lab Chip.

[25] Zhang Y., Lo C.-W., Taylor J.A., Yang S. (2006). Replica molding of high-aspect-ratio polymeric nanopillar arrays with high fidelity. Langmuir.

[26] Rajput D., Costa L., Lansford K., Terekhov A., Hofmeister W. (2013). Solution-cast high-aspect-ratio polymer structures from direct-write templates. ACS Appl. Mater. Interfaces.

[27] Miranda I., Souza A., Sousa P., Ribeiro J., Castanheira E.M.S., Lima R., Minas G. (2022). Properties and applications of PDMS for biomedical engineering: A review. J. Funct. Biomater..

[28] Kotz F., Mader M., Dellen N., Risch P., Kick A., Helmer D., Rapp B. (2020). Fused deposition modeling of microfluidic chips in polymethylmethacrylate. Micromachines.

[29] Mekaru H., Utsumi Y., Hattori T. (2002). Quasi-3D microstructure fabrication technique utilizing hard X-ray lithography of synchrotron radiation. Microsyst. Technol..

[30] Sato H., Houshi Y., Shoji S. (2004). Three-dimensional micro-structures consisting of high aspect ratio inclined micro-pillars fabricated by simple photolithography. Microsyst. Technol..

[31] del Campo A., Arzt E. (2008). Fabrication approaches for generating complex micro-and nanopatterns on polymeric surfaces. Chem. Rev..

[32] Lorenz H., Laudon M., Renaud P. (1998). Mechanical characterization of a new high-aspect-ratio near UV-photoresist. Microelectron. Eng..

[33] Lee J.B., Choi K.-H., Yoo K. (2015). Innovative SU-8 lithography techniques and their applications. Micromachines.

[34] Yoon Y.-K., Park J.-H., Allen M.G. (2006). Multidirectional UV lithography for complex 3-D MEMS structures. J. Microelectromech. Syst..

[35] Jin P. (2004). Ultrathick SU-8 fabrication for microreciprocating engines. J. Micro Nanolithography MEMS MOEMS.

[36] Lee K., Lee H.C., Lee D.-S., Jung H. (2010). Drawing lithography: Three-dimensional fabrication of an ultrahigh-aspect-ratio microneedle. Adv. Mater..

[37] Teh W.H., Dürig U., Salis G., Harbers R., Drechsler U., Mahrt R.F., Smith C.G., Güntherodt H.-J. (2004). SU-8 for real three-dimensional subdiffraction-limit two-photon microfabrication. Appl. Phys. Lett..

[38] Zamfirescu M., Jipa F., Ulmeanu M., Luculescu C., Ionita I., Dabu R. (2009). High-aspect-ratio structures produced by two-photon photopolymerization. J. Optoelectron. Adv. Mater..

[39] Farsari M., Chichkov B.N. (2009). Two-photon fabrication. Nat. Photon.

[40] Kawata S., Sun H.-B., Tanaka T., Takada K. (2001). Finer features for functional microdevices. Nature.

[41] Nielsen A.V., Beauchamp M.J., Nordin G.P., Woolley A.T. (2020). 3D Printed Microfluidics. Annu. Rev. Anal. Chem. Palo. Alto. Calif..

[42] Baldacchini T. (2015). Three-Dimensional Microfabrication Using Two-Photon Polymerization: Fundamentals, Technology, and Applications.

[43] De Volder M., Hart A.J. (2013). Engineering hierarchical nanostructures by elastocapillary self-assembly. Angew. Chem. Int. Ed..

[44] Singh K., Lister J.R., Vella D. (2014). A fluid-mechanical model of elastocapillary coalescence. J. Fluid Mech..

[45] Purtov J., Verch A., Rogin P., Hensel R. (2018). Improved development procedure to enhance the stability of microstructures created by two-photon polymerization. Microelectron. Eng..

[46] Lao Z., Pan D., Yuan H., Ni J., Ji S., Zhu W., Hu Y., Li J., Wu D., Chu J. (2018). Mechanical-tunable capillary-force-driven self-assembled hierarchical structures on soft substrate. ACS Nano.

[47] Chandra D., Yang S. (2010). Stability of high-aspect-ratio micropillar arrays against adhesive and capillary forces. Acc. Chem. Res..

[48] Ghosh T., Fritz E.-C., Balakrishnan D., Zhang Z., Vrancken N., Anand U., Zhang H., Loh N.D., Xu X., Holsteyns F. (2022). Preventing the Capillary-Induced Collapse of Vertical Nanostructures. ACS Appl. Mater. Interfaces.

[49] Xu X., Vrancken N., Vereecke G., Suhard S., Pourtois G., Holsteyns F. (2016). Some critical issues in pattern collapse prevention and repair. Solid State Phenom..

[50] Vrancken N., Vereecke G., Bal S., Sergeant S., Doumen G., Holsteyns F., Terryn H., De Gendt S., Xu X. (2016). Pattern collapse of high-aspect-ratio silicon nanostructures-A parametric study. Solid State Phenom..

[51] Lázár I., Fábián I. (2016). A continuous extraction and pumpless supercritical CO_2_ drying system for laboratory-scale aerogel production. Gels.

[52] Araujo J., Teran F., Oliveira R., Nour E., Montenegro M., Campos J., Vazoller R. (2003). Comparison of hexamethyldisilazane and critical point drying treatments for SEM analysis of anaerobic biofilms and granular sludge. J. Electron Microsc..

[53] Arkles B. (1977). Tailoring surfaces with silanes. Chemtech.

[54] Chandra D., Yang S. (2009). Capillary-force-induced clustering of micropillar arrays: Is it caused by isolated capillary bridges or by the lateral capillary meniscus interaction force?. Langmuir.

[55] Hu Y., Lao Z., Cumming B.P., Wu D., Li J., Liang H., Chu J., Huang W., Gu M. (2015). Laser printing hierarchical structures with the aid of controlled capillary-driven self-assembly. Proc. Natl. Acad. Sci. USA.

[56] Saha S.K., Wang D., Nguyen V.H., Chang Y., Oakdale J.S., Chen S.-C. (2019). Scalable submicrometer additive manufacturing. Science.

[57] Park J.E., Won S., Cho W., Kim J.G., Jhang S., Lee J.G., Wie J.J. (2021). Fabrication and applications of stimuli-responsive micro/nanopillar arrays. J. Polym. Sci..

